# Sexual Difference Matters: Females with High Microsatellite Instability Show Increased Survival after Neoadjuvant Chemotherapy in Gastric Cancer

**DOI:** 10.3390/cancers13051048

**Published:** 2021-03-02

**Authors:** Meike Kohlruss, Katja Ott, Bianca Grosser, Moritz Jesinghaus, Julia Slotta-Huspenina, Alexander Novotny, Alexander Hapfelmeier, Thomas Schmidt, Matthias M. Gaida, Wilko Weichert, Gisela Keller

**Affiliations:** 1 Institute of Pathology, TUM School of Medicine, Technical University of Munich, 81675 Munich, Germany; meike.kohlruss@tum.de (M.K.); bianca.grosser@uk-augsburg.de (B.G.); moritz.jesinghaus@tum.de (M.J.); julia.slotta-huspenina@tum.de (J.S.-H.); wilko.weichert@tum.de (W.W.); 2 Department of Surgery, Klinikum Rosenheim, 83022 Rosenheim, Germany; katja.ott@ro-med.de; 3 Institute of Pathology and Molecular Diagnostics, University Hospital Augsburg, 86156 Augsburg, Germany; 4 Department of Surgery, TUM School of Medicine, Technical University of Munich, 81675 Munich, Germany; alexander.novotny@tum.de; 5 Institute of Medical Informatics, Statistics and Epidemiology, Technical University of Munich, 81675 Munich, Germany; alexander.hapfelmeier@tum.de; 6 Institute of General Practice and Health Services Research, School of Medicine, Technical University of Munich, 81675 Munich, Germany; 7 Department of General, Visceral and Transplantation Surgery, University of Heidelberg, 69120 Heidelberg, Germany; thomas1.schmidt@med.uni-heidelberg.de; 8 Institute of Pathology, University of Heidelberg, 69120 Heidelberg, Germany; matthias.gaida@unimedizin-mainz.de; 9 Institute of Pathology, University Medical Center Mainz, JGU-Mainz, 55131 Mainz, Germany; 10 German Cancer Consortium (DKTK), Partner Site Munich, Institute of Pathology, 81675 Munich, Germany

**Keywords:** adenocarcinoma, gastric, gastroesophageal junction, sex, age, prognosis, neoadjuvant chemotherapy, molecular subtype, microsatellite instability

## Abstract

**Simple Summary:**

Here we report a sex- and age-specific analysis of 717 patients with gastric/gastro-esophageal adenocarcinomas treated with or without neoadjuvant chemotherapy (CTx) regarding overall survival (OS) and response to CTx. The analysis was also performed in molecular subtypes determined previously. Females demonstrated a significantly increased OS particularly in the group of patients treated with neoadjuvant CTx. Specifically in this patient group and taken into account the molecular subtypes, females with high microsatellite instability (MSI-H) showed the best survival followed by the male MSI-H, the female microsatellite stable (MSS) group and the male MSS group. Thus, we show an effect of sex on OS in gastric/gastro-esophageal cancer in particular for patients treated with neoadjuvant CTx. The superior survival of women with MSI-H tumors among the CTx patients implies that the combined consideration of these factors could contribute to an individualized treatment of the patients.

**Abstract:**

We aimed to investigate patients with gastric/gastro-esophageal adenocarcinomas for sex- and age-specific differences regarding overall survival (OS) and response to neoadjuvant chemotherapy (CTx) under consideration of tumor specific molecular subtypes. Overall, 717 patients were analyzed, including 426 patients treated with and 291 treated without neoadjuvant CTx. Microsatellite instability (MSI) and Epstein-Barr virus positivity (EBV+) were determined previously. Females demonstrated a significantly increased OS (*p =* 0.035), particularly in the subgroup treated with CTx (*p =* 0.054). No significant differences regarding age were found. In the molecular subgroups, no sex-related differences were observed in the non-CTx group. However in the CTx group, females with MSI-high (H) tumors showed the best OS (*p =* 0.043), followed by the male MSI-H (*p =* 0.198) and female MSS (*p =* 0.114) compared to the male MSS group as reference. The interaction between sex and MSI in this patient group was noticeable (*p =* 0.053) and was included as a relevant factor in multivariable analyses. In conclusion, our results show an effect of sex on OS in gastric/gastro-esophageal cancer specifically for patients treated with neoadjuvant CTx. The superior survival of women with MSI-H tumors after neoadjuvant CTx implies that combined consideration of these factors could contribute to an individualized treatment of the patients.

## 1. Introduction

Gastric cancer is one of the most common tumors and is still the third leading cause of cancer-related death worldwide [1]. Despite strong efforts in randomized phase III studies and the addition of targeted drugs to established chemotherapy, the prognosis remains poor [2,3,4,5]. In addition, the availability of markers indicating response to specific treatments is still limited. Patient’s sex is a simple, defined, often underestimated and cost-free feature, which is suggested to be a modulator of treatment response and toxicity [6]. In gastric cancer, sex-specific differences are clearly evident in clinical routine. The incidence of this tumor is sex-dependent with male dominance [7]. Gastric cancer of men is more frequently of the intestinal subtype, whereas female sex is associated with the diffuse Lauren type, younger age, signet ring cells, larger tumor size and more advanced T- and N category [8,9,10]. Interestingly, intestinal-type gastric cancer increases in females with time after menopause and the incidence is similar to males after 10 years post menopause, which hints on a hormonal influence on the disease [11]. Regarding survival an improved outcome has been reported for females after surgery and also after neoadjuvant treatment [12,13]. All these data suggest a sex-modulated therapeutic response and prognosis, which in addition may be affected by specific molecular alterations of the tumor.

A molecular classification system of gastric carcinomas has been proposed by The Cancer Genome Atlas (TCGA) project, which encompasses the following subgroups: tumors which are positive for Epstein Barr virus (EBV+), tumors demonstrating microsatellite instability (MSI), tumors with chromosomal instability and genomic stable tumors [14]. Regarding clinical relevance, notably MSI has been shown to be associated with better prognosis, older age, female sex, distal tumor localization and intestinal histological subtype [15,16,17]. MSI is of particular interest as the strong immunogenicity due to the high neoantigen load of these tumor cells making them more susceptible to immunotherapeutic approaches using immune checkpoint inhibitors [18,19,20]. Today, the determination of the MSI status in the context of this treatment is part of daily clinical practice. However, concerning neoadjuvant chemotherapy (CTx), the role of MSI indicating a possible benefit or even harm for these patients is unclear and controversially discussed [21,22,23]. We recently showed that high (H) MSI and EBV (+) indicated a good prognosis, irrespective of treatment with or without CTx in a large cohort of adenocarcinomas of the stomach or the gastro-esophageal junction and that this molecular subgroups were not predictive for response [17].

Knowledge about sex- and age-specific effects related to response and prognosis in the context of platinum/5-FU neoadjuvant chemotherapy in gastric/gastroesophageal cancer is limited. Particularly little is known about predictive or prognostic differences in specific molecular tumor subtypes. Thus, our goal was to fill this gap and we reevaluated our large gastric/gastro-esophageal cohorts and performed a sex and age specific analysis of the cohorts and the molecular subgroups.

## 2. Materials and Methods

### 2.1. Patients

Tumors from 717 patients with gastric adenocarcinomas (GC) including tumors of the gastro-esophageal junction (AEG II and AEG III according to Siewert and Stein) [24] that were treated with (*n* = 426) or without neoadjuvant (*n* = 291) CTx between 2001 and 2013 at the Department of Surgery of the University of Heidelberg and between 1993 and 2012 at the Technical University of Munich were included. The indication for neoadjuvant CTx was as described in detail [25,26]. In brief, eligibility for neoadjuvant CTx included the presence of locally advanced adenocarcinoma (cT3-T4, any N, cM0 by endoscopy, endoluminal ultrasound and CT scan). Patient characteristics of the CTx and non-CTx group have been described in detail previously indicating that among the 291 patients who underwent primary surgery 128 patients with cT2 and 162 patients with cT3/cT4 tumors were included. For one patient these data were not available [17]. The cT3/cT4 group treated with surgery alone refers to patients who refused chemotherapy or were not treated with CTx due to individual treatment decisions or relevant comorbidities.

Molecular subgroups have been determined in a recent study [17]. Regarding patients treated with neoadjuvant CTx, the determination of the molecular subgroups was based on the analysis of 100 pretherapeutic biopsies before CTx and 284 resected tumors after CTx. For additional 42 patients, corresponding biopsies before and resected tumors after CTx had been analyzed in that previous study and no differences regarding MSI and EBV status were identified [17]. In respect to MSI, there were 4 patients with MSI-H and 38 patients with MSS in the biopsies and consistent classification in the corresponding resected tumors. Thus for the purpose of the present study we combined the patients treated with neoadjuvant CTx into one group encompassing tumors from an overall 426 patients.

**Figure 1:**
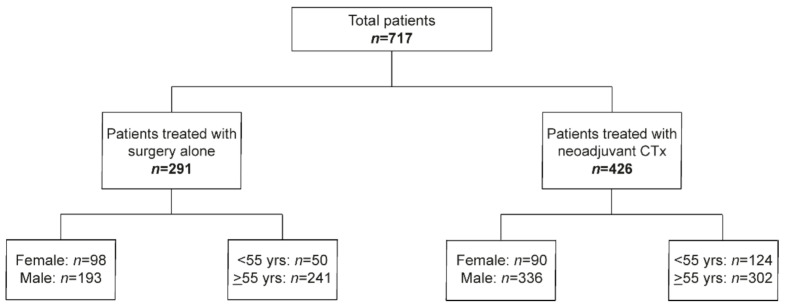
Overview of the study.

For age dichotomization, the cut off at 55 years was chosen to address possible differences regarding the pre- and postmenopausal stage. The study population including the number of males and females and the number of patients <55 and ≥55 years in the GC cohort without and after CTx is shown in Figure 1.

### 2.2. Chemotherapy

The chemotherapeutic regimens stratified according to sex and age are detailed in Table S1.

### 2.3. Response Evaluation

Response to neoadjuvant CTx was evaluated as described [17,25]. In brief, tumor regression was determined histopathologically and was classified into three tumor regression grades (TRG): TRG1, TRG2 and TRG3, which corresponded to <10%, 10–50% and >50% residual tumor cells within the tumor bed respectively. Patients with TRG1 were classified as responders, while those with TRG2 and TRG3 as non-responders.

### 2.4. Follow-Up and Overall Survival

Follow-up was performed as described and the median follow-up period of all 717 patients was 60.7 months (95% CI, 56.5–64.9). Overall survival (OS) was defined as the time between the date of surgery and death by any cause [17,27].

### 2.5. Molecular Analysis

The molecular analyses including DNA isolation, analysis and definition of MSI and detection of EBV are described in detail in our previous study [17]. In brief, DNA from normal and tumor tissue had been isolated from formalin fixed paraffin embedded (FFPE) tissues after manual microdissection. MSI was analyzed using the Bethesda panel consisting of two mononucleotide repeat marker BAT25 and BAT26 and three dinucleotide repeat marker D2S123, D5S346 and D17S250 as recommended by the National Cancer Institute [28]. According to a standardized definition, MSI-H was defined if at least two of the five markers showed MSI corresponding to at least 40% of unstable markers and as low (L) MSI, if one of the five markers showed MSI. Due to the high sensitivity and specificity of mononucleotide repeat markers to detect MSI-H and thus to avoid a classification as MSI-H based on instabilities exclusively at two dinucleotide repeats, those tumors were additionally analyzed using three mononucleotide markers NR-21, NR-24 and NR-27 as described in detail in supplementary material [29,30]. If no instabilities at these mononucleotide repeats were observed, the tumors were reclassified as MSI-L. Tumors without MSI were classified as microsatellite stable (MSS).

Screening for EBV was performed by a PCR-based assay and in situ hybridization for selected cases as described [17].

### 2.6. Statistical Analysis

Chi-squared tests or Fisher’s exact tests were used for hypothesis testing of differences between relative frequencies. Kaplan–Meier estimates of survival probabilities were compared by log rank tests. Relative risks were estimated by hazard ratios (HRs) from Cox proportional hazards models. Multivariable analysis was performed by stepwise forward variable selection using Wald tests. Interaction effects of MSI and sex were included into the model formulas to explore sex-specific effects of MSI. Statistical analyses were performed using SPSS, Version 25 (IBM Corp., Armonk, NY, USA). Exploratory 5% significance levels (two-tailed) were used for hypothesis testing.

## 3. Results

### 3.1. Sex/Age and Association with Patient Characteristics and Response to Chemotherapy

Overall, the GC cohort consisted of 188 (26%) females and 529 (74%) males and 174 (24%) patients <55 years and 543 (76%) patients ≥55 years. Patients’ characteristics are summarized in detail in Table 1. Tumors of intestinal type and with proximal location were significantly more frequently found in males compared to females (each *p* < 0.001). Younger age (<55 years) was associated with non-intestinal tumor type (*p* = 0.007) and metastasis (*p* = 0.040). Furthermore, younger patients and men were more frequently treated with CTx (each *p* < 0.001) (Table 1). Subgroup analysis stratified by sex and age (< and ≥55 years) revealed that women with non-intestinal-type tumors were significantly younger than women with intestinal type tumors (*p* = 0.027) (Table S2).

The question if sex or age may be associated with response to neoadjuvant treatment in terms of tumor regression was assessed in the 426 patients treated with neoadjuvant CTx including 45 (11%) responding (TRG1) and 381 (89%) non-responding patients (TRG2/3). No association with sex or age was found (*p* = 0.336 and *p* = 0.314 respectively) (Table 1).

### 3.2. Sex/Age and Survival of the Patients

**Figure 2:**
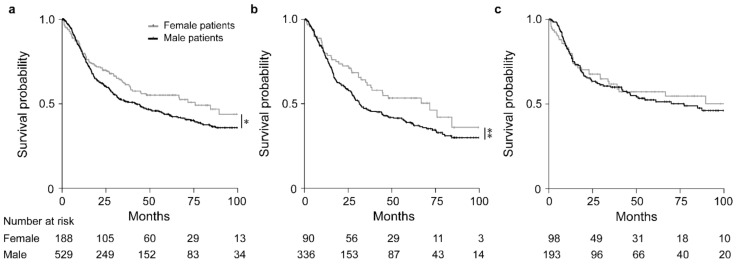
Discrimination of patients’ survival by sex. Kaplan–Meier curves of female or male patients are shown. All GC patients (**a**), patients treated with neoadjuvant CTx (**b**) and patients treated with surgery alone (**c**). * *p =* 0.035, ** *p =* 0.054, Cox-regression.

Overall, females had a significantly increased OS in comparison to males (HR, 0.77; 95% CI, 0.60–0.99, *p* = 0.035) (Figure 2a). Subgroup analysis in the groups stratified by CTx yes/no, revealed that in particular females demonstrated an increased survival after neoadjuvant CTx (HR, 0.72; 95% CI, 0.51–1.01, *p* = 0.054) (Figure 2b), whereas no significant difference between males and females was found in the group treated with surgery alone (HR, 0.90; 95% CI, 0.61–1.32, *p* = 0.587) (Figure 2c). Survival data are summarized in Table 2. As patients with cT2 tumors were included in the group treated with surgery alone, we performed a sex-specific analysis also in the patients stratified according to cT2 and cT3/cT4 showing no significant sex-related differences in both groups (Figure S1a,b).

Regarding age, no significant differences were observed in the study as a whole nor in the patient subgroup treated with CTx. Younger patients (<55 yrs) treated with surgery alone showed a somewhat increased OS, but the difference was not statistically significant (*p* = 0.106) (Table S3 and Figure S2).

### 3.3. Sex/Age and Frequency of Molecular Subgroups and Association with Patient Characteristics and Response to Chemotherapy

Concerning the frequencies of the molecular subgroups, EBV(+) was found in 27/529 (5.1%) males and in 2/188 (1.1%) females, a difference which was statistically significant (*p* = 0.016). MSI-H was found in 45/529 (8.5%) males compared to 22/188 (11.7%) females and the MSI-L phenotype was observed in 30/529 (5.7%) men and 7/188 (3.7%) women (overall *p* = 0.278). Regarding age, MSI-H was significantly associated with older age (*p* = 0.004) and EBV(+) occurred more often at younger age (*p* = 0.080). Data are included in Table 1. All MSI-H tumors were negative for EBV.

Out of the overall 67 tumors classified as MSI-H in this study, 66 tumors showed MSI at least at 3 of the 5 markers of the Bethesda panel with all of them demonstrating instability at least at one of the mononucleotide repeat marker BAT25 or BAT26. One MSI-H tumor showed instabilities at BAT25 and BAT26 and at the three mononucleotide markers additionally tested in this case. The frequencies of MSI at each marker of the Bethesda panel are shown in Figure S3. Examples of MSI at mono- and dinucleotide repeat markers are shown in Figure S4.

Due to the general low frequencies of EBV(+) and MSI-L tumors and of the low frequency of MSI-H tumors specifically in younger patients, further analyses for an association with response to CTx in terms of tumor regression and with patient’s survival were only performed for the MSI-H and microsatellite stable (MSS)/EBV(−) subgroups in relation to sex (Table 1). This corresponded to 45 males and 22 females with MSI-H tumors and to 454 males and 159 females with MSS/EBV(−) tumors.

No association with response to CTx was shown for the sex-specific MSI-H and MSS/EBV(−) subgroups (*p* = 0.330) (Table S4).

### 3.4. Sex-Specificity of the Molecular Subgroups and Survival of the Patients

**Figure 3:**
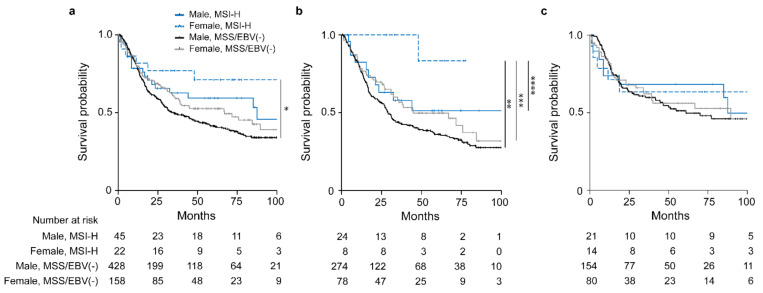
Discrimination of patients’ survival by sex and MSI status. Kaplan–Meier curves of female or male patients with MSI-H or MSS/EBV(−) tumors are shown. All GC patients (**a**), patients treated with neoadjuvant CTx (**b**) and patients treated with surgery alone (**c**). * *p* = 0.024, ** *p* = 0.043, *** *p* = 0.085, **** *p* = 0.125, Cox regression was used to compare the MSI-H female group to the other sex specific molecular subgroups.

Comparison of OS between males and females with the respective MSI-H and MSS/EBV(−) tumors revealed a statistically significant difference (overall *p* = 0.031). The female MSI-H group showed the best OS (HR, 0.39; 95% CI, 0.18–0.89, *p* = 0.024) followed by the male MSI-H group (HR, 0.69; 95% CI, 0.43–1.11, *p* = 0.126) and the female MSS/EBV(−) group (HR, 0.79; 95% CI, 0.61–1.04, *p* = 0.095) taking the male MSS/EBV(−) group as reference (Figure 3a, Table 3).

Subgroup analysis of patients stratified whether they received CTx or not revealed an obvious survival difference for the patients treated with neoadjuvant CTx (overall *p* = 0.056) (Figure 3b). Of note, this difference was mainly due to the striking good OS of the female MSI-H group (HR, 0.13; 95% CI, 0.02–0.94, *p* = 0.043) followed by the male MSI-H group (HR, 0.66; 95% CI, 0.35–1.25, *p* = 0.198) and the female MSS group (HR, 0.75; 95% CI, 0.53 1.07, *p* = 0.114) with the male MSS/EBV(−) group as reference (Figure 3b). No significant differences were observed for the patients treated with surgery alone (overall *p* = 0.898) (Figure 3c). All survival data are summarized in Table 3. In addition, no significant differences were shown in the non-CTx group stratified according to cT2 and cT3/cT4 (Figure S1c,d).

**Figure 4:**
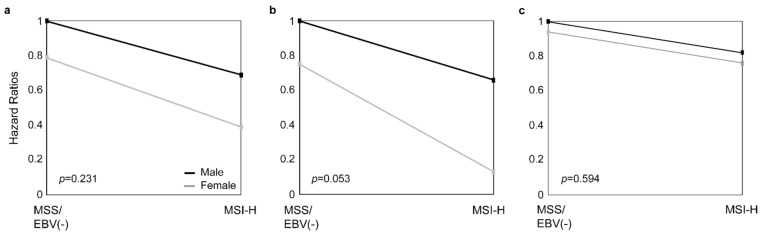
Interaction between sex and MSI status. The interaction between sex and the MSI status by Cox regression models is shown for all GC patients (**a**), patients treated with neoadjuvant CTx (**b**), and patients treated with surgery alone (**c**).

Testing for an interaction between sex and MSI status by Cox regression models was specifically noticeable in the subgroup of patients treated with CTx (*p* = 0.053) (Figure 4).

### 3.5. Multivariable Analysis

Multivariable analysis including the interaction of sex and MSI status as a factor and adjusting for pretherapeutically available variables (age, cT, tumor localization, histological type, CTx yes/no) revealed cT (*p* < 0.001) and the MSI status (*p* = 0.011) as significant independent prognostic factors (Table S5). Including the post-therapeutically available factors as variables (age, (y)pT, (y)pN, R-, M-status, tumor localization, histological type, CTx yes/no) revealed (y)pN (*p* < 0.001), M-status (*p* < 0.001), R-status (*p* < 0.001), tumor localization (*p* = 0.006) and (y)pT (*p* = 0.010) as significant independent prognostic factors (Table S5). 

Multivariable analysis adjusting for pretherapeutically available factors in the subgroups of patients stratified according to CTx yes/no demonstrated the interaction of sex and MSI status as a prognostic factor in the CTx group (*p* = 0.051) (Table S6). Adjusting for posttherapeutically available factors, the interaction was also included as a relevant variable (*p* = 0.067) after R-category (*p* = 0.001), ypN (*p* < 0.001), M-status (*p* < 0.001), and ypT (*p* = 0.019) in this group (Table S6).

## 4. Discussion

There is increasing evidence that sex-specific characteristics affect the success of specific therapeutic interventions, but in oncology the impact of sex on all types of efficacy, toxicity and response has rarely systematically been investigated so far. In phase III studies females are usually underrepresented (37%) and the vast majority (64%) of studies do not report results differentiated by sex [31].

In the study presented here, we performed a comprehensive sex- and age-based analysis of large gastric cancer cohorts treated with and without neoadjuvant chemotherapy. In addition, we investigated if molecular subgroups showed sex- or age-related differences regarding prognosis and response to neoadjuvant CTx.

In our study as a whole, females showed an increased OS, which was particularly evident in the group treated with CTx. An increased OS of female GC patients has been recently reported in a metaanalysis of four prospective randomized trials investigating the use of neoadjuvant CTx in gastric cancer, which essentially is in line with our study [13]. In addition, an increased OS for women receiving platinum-based chemotherapy has been reported for patients with adenocarcinomas of the lung and for adjuvant CTx of colorectal cancer patients, suggesting a general better response to various chemotherapeutic approaches of the female sex in different tumor entities [6,32,33]. The reasons for these findings may be manifold. Sex is a biological variable, which leads to physiological and anatomical differences and there is increasing evidence that there are considerable sex-based variations in pharmacodynamics and pharmacokinetics [6,34]. Platinum compounds mainly exhibit their cytotoxic effect by producing inter- and intrastrand DNA adducts and differences in the DNA damage response encompassing homologous recombination-related dependent and independent mechanisms are supposed to be critical for drug response [35]. However, chemotherapeutic agents as platinum-based compounds not only exhibit direct cytotoxic effects on the tumor cells, but also can induce alterations of the microenvironment of the tumor and it has been shown that a specific immune contexture can affect prognosis and response to therapy [36,37,38,39]. Of note, differences in immune responses, affecting both the innate and adaptive immune system, exist with women demonstrating stronger immune reactions than men [40].

The most interesting finding of our study was the extremely good OS of female patients with the MSI-H phenotype in their tumors after neoadjuvant CTx. In addition, the interaction between sex and MSI status emerged as a relevant prognostic factor in multivariable analysis including pre- and posttherapeutically available variables in the group of patients treated with neoadjuvant CTx. Thus, this emphasizes the importance of consideration of both a patient-specific features and the molecular genetic properties of the tumor augmenting an additional layer of complexity related to the sex-based prognostic differences as observed in our study. A prognostic relevance of the MSI-H phenotype has been recognized for a long time and has been reported in various studies including gastric carcinomas [15,17,41,42]. This has been related to the presentation of multiple neoantigens on the tumor cell surface due to the DNA mismatch repair deficiency of MSI tumors leading to increased immunogenic properties and an increase in infiltrating lymphocytes in MSI-H tumors has been described in various studies [43,44].

Thus, taken altogether, one can speculate that the good OS of the female MSI-H group after CTx, may at least partly be due to an immunogenic boosting effect of CTx, which may be stronger in an already immunogenic “hot” MSI-H tumor of women, who additionally are suggested to have stronger immune responses than men [36,39,40].

Furthermore, we did not find a correlation of the sex-specific molecular subgroups with response to therapy in terms of measurable tumor regression. Specific immune-related response criteria (irRC) have been established for monitoring response to therapies targeting the immune system and among others, a delay in time of a clinical effect has been described for these therapies [45]. Thus, it is tempting to speculate that the lack of correlation with tumor regression in our study may be due to specific differences in response patterns of a primarily cytotoxic and an immune-based effect of a given therapeutic agent.

Beyond that, tumor-specific mutation patterns or sex-biased genetic signatures have been described, which may also contribute to the prognostic differences between females and males [46]. In a previous study, we showed a significant association of p53 mutations with the male sex and patients with mutated p53 and a MSI-H tumor showed a worse survival when treated with neoadjuvant CTx [47].

We believe that our findings may be particularly relevant in the context of the current discussion about the therapeutic treatment of gastric cancer patients with MSI-H tumors using immune checkpoint inhibitors (ICI) [22,23]. MSI has been identified as a biomarker indicating good response to ICI treatment including gastric carcinomas [18,19,20,48]. With respect to neoadjuvant CTx, the MAGIC trial conversely showed a worse OS of MSI-H patients treated with CTx compared to those treated with surgery alone, suggesting that neoadjuvant CTx may even harm patients with MSI-H tumors [21,41]. However, the number of MSI-H tumors was small and no sex-differentiated analysis was performed in that study [21]. Based on our previously published results [17] and the data presented here, a general recommendation not to treat patients with MSI-H tumors with neoadjuvant CTx is not justified. Sex should be taken into consideration as much MSI-H females might gain substantial benefit from this type of therapy, whereas a higher efficacy using immunotherapeutic approaches has been reported for men in a recent meta-analysis including different tumor types [49].

Regarding age, we only found small differences of OS in respect to treatment with or without CTx, which is line with previous studies and suggests that age alone might not be an appropriate therapeutic selection criterion [13]. However, an age cut mimicking the menopause seems strongly to determine the histotype of gastric cancer in females [50].

We are aware that our study has limitations. These refer mainly to its retrospective nature and thus our study has to be considered an explorative analysis. Furthermore, although our overall cohort is large, the consideration of specific subgroups leads to an analysis of a small number of patients in some cases and thus should be interpreted with care. Another limitation refers to the fact that our patients were not treated in the frame of a randomized clinical trial, but rather reflects patients’ treatment in a real world situation. However, the association of sex- and age-based differences with patients characteristics, which we found in this and our previous study [17], as an association of intestinal type tumors with males and the preferential occurrence of non-intestinal tumors in younger females, are in line with reports by others and underscore the representative features and composition of our gastric cancer cohorts [11,50,51].

## 5. Conclusions

Our sex- and age-based analysis reveals remarkable differences specifically between men and women with an obvious increased survival of females after neoadjuvant chemotherapy, which was particularly evident for females with an MSI-H tumor. This implies that the combined consideration of sex and specific molecular characteristics of the tumor could contribute to choose the adequate treatment for each individual patient. Further studies including sex- and age-specific evaluations in conjunction with adequate molecular analysis are mandatory. The participation of females in clinical studies evaluating specific therapeutic options should be encouraged since females are often underrepresented and a sex-based analysis should be recommended for every study in the future.

## Figures and Tables

**Table 1:** Clinical-pathological characteristics of the patients stratified according to sex and age.

Category	Value	Female *n* (%)	Male *n* (%)	*p*-Value ^1^	<55 Years *n* (%)	≥55 Years *n* (%)	*p*-Value ^1^
Cases	Total	188 (100)	529 (100)		174 (100)	543 (100)	
Tumor localization	Proximal	75 (39.9)	298 (56.3)	0.001	94 (54.0)	279 (51.4)	0.581
	Middle	52 (27.7)	116 (21.9)		39 (22.4)	129 (23.7)	
	Distal	47 (25.0)	92 (17.4)		36 (20.7)	103 (19.0)	
	Total/linitis	14 (7.4)	16 (3.6)		5 (2.9)	28 (5.2)	
	n/a	-	4 (<1)		-	4 (<1)	
Proximal versus non-proximal	Proximal	75 (39.9)	298 (56.3)	<0.001	94 (54.0)	279 (51.4)	0.604
	Non-proximal	113. (60.1)	227 (42.9)		80 (46.0)	260 (47.9)	
	n/a	-	4 (<1)		-	4 (<1)	
Laurén classification	Intestinal	82 (43.6)	315 (59.5)	<0.001	81 (46.6)	316 (58.2)	0.007
	Non-intestinal	106 (56.4)	214 (40.5)		93 (53.4)	227 (41.8)	
Tumor grade	G1/2	36 (19.1)	111 (21.0)	0.485	27 (15.5)	120 (22.1)	0.061
	G3/4	131 (69.7)	347 (65.6)		124 (71.3)	354 (65.2)	
	n/a	21 (11.2)	71 (13.4)		23 (13.2)	69 (12.7)	
Clinical tumor stage	cT2	46 (24.5)	103 (19.5)	0.123	29 (16.7)	120 (22.1)	0.135
	cT3/cT4	138 (73.4)	422 (79.8)		142 (81.6)	418 (77.0)	
	n/a	4 (2.1)	4 (<1)		3 (1.7)	5 (<1)	
(y)pT ^2^	(y)pT0	5 (2.7)	4 (0.8)	0.030	4 (2.3)	5 (0.9)	0.293
	(y)pT1	19 (10.1)	48 (9.1)		14 (8.1)	53 (9.8)	
	(y)pT2	24 (12.8)	70 (13.2)		17 (9.8)	77 (14.2)	
	(y)pT3	85 (45.2)	297 (56.1)		99 (56.9)	283 (52.1)	
	(y)pT4	53 (28.2)	110 (20.8)		39 (22.4)	124 (22.8)	
	n/a	2 (1.1)	-		1 (<1)	1 (<1)	
(y)pN ^2^	Negative	60 (31.9)	179 (33.8)	0.695	48 (27.6)	191 (35.2)	0.069
	Positive	126 (67.0)	350 (66.2)		125 (71.8)	351 (64.6)	
	n/a	2 (1.1)	-		1 (<1)	1 (<1)	
Metastasis status	No	156 (83.0)	442 (83.6)	0.920	136 (78.2)	462 (85.1)	0.040
	Yes	30 (15.9)	87 (16.4)		37 (21.3)	80 (14.7)	
	n/a	2 (1.1)	-		1 (<1)	1 (<1)	
Resection category	R0	148 (78.7)	402 (76.0)	0.319	130 (74.7)	420 (77.3)	0.524
	R1	38 (20.2)	127 (24.0)		43 (27.7)	122 (22.5)	
	n/a	2 (1.1)	-		1 (<1)	1 (<1)	
Neoadjuvant CTx	No	98 (52.1)	193 (36.5)	<0.001	50 (28.7)	241 (44.4)	<0.001
	Yes	90 (47.9)	336 (63.5)		124 (71.3)	302 (55.6)	
Response ^3^	Responder (TRG1)	12 (13.3)	33 (9.8)	0.336	16 (12.9)	29 (9.6)	0.314
	Non-responder (TRG2/3) ^4^	78 (86.7)	303 (90.2)		108 (87.1)	273 (90.4)	
	Total ^3^	90 (100)	336 (100)		124 (100)	302 (100)	
MSI status	MSS	159 (84.6)	454 (85.8)	0.278	162 (93.1)	451 (83.1)	0.004
	MSI-L	7 (3.7)	30 (5.7)		5 (2.9)	32 (5.9)	
	MSI-H	22 (11.7)	45 (8.5)		7 (4.0)	60 (11.0)	
EBV status	EBV negative	186 (98.9)	502 (94.9)	0.016	163 (93.7)	525 (96.7)	0.080
	EBV positive	2 (1.1)	27 (5.1)		11 (6.3)	18 (3.3)	

CI, confidence interval; TRG, tumor regression grade, CTx, chemotherapy; MSS, microsatellite stable; MSI-L, low microsatellite instability; MSI-H, high microsatellite instability; EBV, Epstein–Barr virus; ^1^ Chi-squared test; ^2^ TNM classification according to 7th Edition UICC; ^3^ Response to neoadjuvant treatment in terms of tumor regression corresponded only to patients with tumors treated with neoadjuvant CTx. ^4^ Two patients with tumor progression during CTx were not operated and classified as TRG3 and as non-responders respectively.

**Table 2:** Survival data of the patient cohort and subgroups in association with sex.

Patient Cohort and Subgroups	Sex	No.	Events	Survival Probability (%)			Median Survival (Months)	HR	*p*-Value ^1^
				1 Year	3 Years	5 Years	(95% CI)	(95% CI)	
All tumor specimens	Female	188	78	79.7	61.4	55.2	75.8 (42.6–109.0)	0.77 (0.60–0.99)	0.035
	Male	529	266	78.4	51.4	43.8	41.7 (29.8–53.6)	1 ref.	-
	Total	717	344	78.8	54.0	46.8	46.3 (33.1–59.5)	-	-
Tumors with neoadjuvant CTx	Female	90	41	79.8	61.1	53.4	71.6 (42.4–100.9)	0.72 (0.51–1.01)	0.054
	Male	336	181	77.7	46.4	38.9	31.4 (22.1–40.7)	1 ref.	-
	Total	426	222	78.1	49.7	42.0	35.9 (27.2–44.6)	-	-
Tumors without neoadjuvant CTx	Female	98	37	79.8	61.9	57.2	nr	0.90 (0.61–1.32)	0.587
	Male	193	85	79.8	59.9	52.4	77.3 (36.7–117.9)	1 ref.	-
	Total	291	122	79.7	60.5	54.0	85.0 (51.7–118.3)	-	-

CI, confidence interval; HR; Hazard ratio; ref., reference; nr, not reached; ^1^ Cox-regression.

**Table 3:** Survival data of the patient cohort and subgroups stratified according to sex of the patients in association with the MSI status.

Patient Cohort and Subgroups	Sex and MSI Status	No.	Events	Survival Probability (%)			Median Survival (Months)	HR	*p*-Value ^1^
				1 Year	3 Years	5 Years	(95% CI)	(95% CI)	
All tumor specimens									0.031
	Female, MSI-H	22	6	81.8	77.0	71.1	nr	0.39 (0.18–0.89)	0.024
	Female, MSS/EBV(−)	158	69	79.7	59.1	52.6	66.9 (33.9–99.9)	0.79 (0.61–1.04)	0.095
	Male, MSI-H	45	18	78.5	62.6	59.3	87.5 (-)	0.69 (0.43–1.11)	0.126
	Male, MSS/EBV(−)	428	222	78.5	49.3	41.4	33.8 (24.1–43.5)	1 ref.	-
	Total ^2^	653	315	78.9	53.6	46.5	45.3 (31.5–59.1)	-	-
Tumors with neoadjuvant CTx									0.056
	Female, MSI-H	8	1	100	100	83.3		0.13 (0.02–0.94)	0.043
	Female, MSS/EBV(−)	78	39	78.1	56.7	49.7	44.9 (14.2–75.6)	0.75 (0.53–1.07)	0.114
	Male, MSI-H	24	10	82.4	57.7	51.3	nr	0.66 (0.35–1.25)	0.198
	Male, MSS/EBV(−)	274	156	76.3	43.4	36.1	29.1 (25.2–33.0)	1 ref.	-
	Total ^2^	384	206	77.8	48.5	40.9	33.8 (25.5–42.1)	-	-
Tumors without neoadjuvant CTx									0.898
	Female, MSI-H	14	5	71.4	63.5	63.5	nr	0.76 (0.30–1.88)	0.548
	Female, MSS/EBV(−)	80	30	81.7	62.1	56.1	89.5 (-)	0.94 (0.61–1.45)	0.772
	Male, MSI-H	21	8	73.9	68.2	68.2	87.5 (-)	0.82 (0.39–1.72)	0.604
	Male, MSS/EBV(−)	154	66	81.7	59.9	51.2	61.1 (25.5–96.7)	1 ref.	-
	Total ^2^	269	109	80.5	61.5	54.9	87.5 (-)	-	-

HR; Hazard ratio; CI, confidence interval; ref., reference; nr, not reached; MSI-H, high microsatellite instability; MSS, microsatellite stable; EBV(−), Epstein-Barr virus negative; ^1^ Cox-regression; ^2^ Only patients with MSI-H and MSS/EBV(−) tumors were included in this analysis.

## Data Availability

The data presented in this study are available in this article and supplementary material.

## Supplementary Materials

The following are available online at https://www.mdpi.com/2072-6694/13/5/1048/s1, Supplementary Methods; Table S1: Chemotherapy regimens of the preoperatively treated patients stratified according to sex and age, Table S2: Subgroup analysis in male and female patients stratified according to age and histological subtypes, Table S3: Survival data of the patient cohort and subgroups in association with age, Table S4: Response to neoadjuvant CTx regarding the sex specific MSI-H and MSS/EBV(−) groups, Table S5: Multivariable analysis of survival including the interaction of sex and MSI and pre- and posttherapeutically available clinical factors in all MSI-H and MSS/EBV(−) patients (*n* = 653), Table S6: Multivariable analysis of survival including the interaction of sex and MSI and pre- and posttherapeutically available clinical factors in MSI-H and MSS/EBV(−) patients treated with neoadjuvant CTx (*n* = 384), Figure S1: Patients’ survival in the non-CTx group stratified according to clinical tumor stage, Figure S2: Discrimination of patients’ survival by age, Figure S3: Frequency of instabilities at five microsatellite markers included in the Bethesda panel in MSI-H tumors (*n* = 67), Figure S4: Capillary electrophoresis results of MSI analysis using the five microsatellite markers included in the Bethesda panel.
