# Supplementary material for: Sexual Difference Matters: Females with High Microsatellite Instability Show Increased Survival after Neoadjuvant Chemotherapy in Gastric Cancer
Source: Cancers (Basel). 2021 Mar 2;13(5):1048. doi: 10.3390/cancers13051048 (PMC7958600; doi:10.3390/cancers13051048)
Supplement: Supplementary file 1 [file cancers-13-01048-s001.pdf]

Supplementary Materials

Sexual difference matters: females with high microsatellite instability show increased survival after neoadjuvant chemotherapy in gastric cancer

Meike Kohlruss, Katja Ott, Bianca Grosser, Moritz Jesinghaus, Julia Slotta-Huspenina, Alexander Novotny, Alexander Hapfelmeier, Thomas Schmidt, Matthias M. Gaida, Wilko Weichert and Gisela Keller

Supplementary Methods

MSI analysis using mononucleotide repeats

MSI analysis using the three mononucleotide repeats NR-21, NR-24, NR-27 was performed in a multiplex PCR with fluorescence-tagged primers using the Type-it Microsatellite PCR kit according to the instructions of the manufacturer (Qiagen, Hilden, Germany). Primer sequences were as published [30] and cycle conditions were as follows: after an initial step of 95°C for 5 min, 32 cycles were performed consisting of denaturation at 95°C for 30 sec, annealing at 58°C for 90 sec and extension at 72°C for 30 sec and final extension at 60°C for 30 min. Separation and detection of the PCR products were performed in a 3130 Genetic Analyzer (Applied Biosystems, Foster City, CA) as described previously [17].

Table S1. Chemotherapy regimens of the preoperatively treated patients stratified according to sex and age.

Neoadjuvant Chemotherapy	Female		Male		<55 years		≥55 years	
	n	%	*n*	%	*n*	%	*n*	%
Total	90	100	336	100	124	100	302	100
Cis + 5-FU or Cap	49	54.5	164	48.8	65	52.4	148	49.1
Ox + 5-FU or Cap	11	12.2	40	11.9	3	2.4	48	15.9
Cis + 5-FU + Doc or Pac	5	5.6	24	7.1	16	12.9	13	4.3
Ox + 5-FU + Doc	4	4.4	17	5.1	10	8.1	11	3.6
Cis or Ox + 5-FU or Cap + Epi	13	14.4	75	22.3	23	18.6	65	21.5
Others	7	7.8	16	4.8	7	5.6	16	5.3
n/a	1	1.1	-	-	-	-	1	<1

CTx, neoadjuvant chemotherapy; Cis, cisplatin; Ox, oxaliplatin; 5-FU, 5-fluorouracil; Cap, capecitabine; Doc, docetaxel; Pac, paclitaxel; Epi, epirubicin; Others, combination of Cis/Ox with other agents or cross over between different treatment regimens; n/a, no data available.

Table S2. Subgroup analysis in male and female patients stratified according to age and histological subtypes.

	Age	Intestinal Subtype *n* (%)	Non-Intestinal Subtype *n* (%)	*p*-Value [1]
Male patients	<55 years	67 (21.3)	60 (28.0)	0.074
	≥55 years	248 (78.7)	154 (72.0)	
	Total	315 (100)	214 (100)	
Female patients	<55 years	14 (17.1)	33 (31.1)	0.027
	≥55 years	68 (82.9)	73 (68.9)	
	Total	82 (100)	106 (100)	

[1]Chi-squared test.

Table S3. Survival data of the patient cohort and subgroups in association with age.

Patient Cohort and Subgroups	Age	No.	Events	Survival Probability (%)			Median Survival (months)	HR	*p*-Value [1]
				1 yr	3 yrs	5 yrs	(95% CI)	(95% CI)	
All tumor specimens	<55 yrs	174	84	82.7	56.5	46.4	51.0	0.93	0.555

							(30.6–71.4)	(0.73–1.19)	
	≥55 yrs	543	260	77.5	53.3	47.0	46.3 (30.4–62.2)	1 ref.	-
	Total	717	344	78.8	54.0	46.8	46.3 (33.1–59.5)	-	-
Tumors with neoadjuvant CTx	<55 yrs	124	67	80.5	50.4	42.0	38.0 (21.0–55.1)	1.0 (0.75–1.34)	0.988
	≥55 yrs	302	155	77.2	49.5	42.0	35.6 (24.6–46.6)	1 ref.	-
	Total	426	222	78.1	49.7	42.0	35.9 (27.2–44.6)	-	-
Tumors without neoadjuvant CTx	<55 yrs	50	17	88.6	71.8	57.7	nr	0.66 (0.39–1.10)	0.106
	≥55 yrs	241	105	77.9	58.3	53.5	77.3 (43.1–111.5)	1 ref.	-
	Total	291	122	79.7	60.5	54.0	85.0 (51.7–118.3)	-	-

HR, Hazard ratio; CI, confidence interval; ref., reference; nr, not reached; [1] Cox-regression.

Table S4. Response to neoadjuvant CTx regarding the sex specific MSI-H and MSS/EBV(–) groups.

Category	Value	Male, MSS/EBV(–) *n* (%)	Male, MSI-H *n* (%)	Female, MSS/EBV(–) *n* (%)	Female, MSI-H *n* (%)	*p*-Value [1]
Response to neoadjuvant CTx	Responder (TRG1)	24 (8.8)	2 (8.3)	9 (11.5)	2 (25)	0.330
	Non-responder [2] (TRG2/3)	250 (91.2)	22 (91.7)	69 (88.5)	6 (75)	
Total		274 (100)	24 (100)	78 (100)	8 (100)	

TRG, tumor regression grade; CTx, chemotherapy; [1] Fisher's exact test; [2] Two patients with tumor progression during CTx were not operated and classified as TRG3 and as non-responders respectively.

Table S5. Multivariable analysis of survival including the interaction of sex and MSI status and pre- and posttherapeutically available clinical factors in all MSI-H and MSS/EBV(-) patients (n=653).

		HR	95% CI	*p*-Value [1]
**All patients: Pretherapeutic factors [2]**				
	**Clinical tumor stage**			
	cT2	1 ref.	-	<0.001
	cT3/4	2.60	1.86-3.65	
	**MSI status**			
	MSS/EBV(–)	1 ref.	-	0.011
	MSI-H	0.58	0.38-0.88	
**All patients: Posttherapeutic factors [3]**				
	**(y)pN [4]**			
	(y)pN0	1 ref.	-	<0.001
	(y)pN1	2.40	1.77-3.25	
	**Metastasis status**			
	Negative	1 ref.	-	<0.001
	Positive	1.87	1.43-2.45	
	**R-category**			
	R0	1 ref.	-	<0.001
	R1	1.64	1.26-2.13	
	**Localization**			
	Proximal	1 ref.	-	0.006
	Non-proximal	0.72	0.58-0.91	
	**(y)pT [4]**			0.010
	(y)pT0	0.20	0.03-1.46	0.113
	(y)pT1	0.40	0.20-0.77	0.006
	(y)pT2	0.56	0.36-0.87	0.010

(y)pT3	0.74	0.56-0.97	0.028
(y)pT4	1 ref.	-	-

ref., reference; MSS, microsatellite stable; EBV(-), Epstein-Barr virus negative; MSI-H, high microsatellite instability, HR; Hazard ratio; CI, confidence interval. [1] Wald-Test of Hazard Ratio; [2] Pre-therapeutic factors include: Sex, age (≥55 vs <55 yrs), localization (proximal vs non-proximal), Laurén subtypes (intestinal vs non-intestinal), neoadjuvant chemotherapy (yes vs no), clinical tumor stage (cT2 vs cT3/4), MSI status (MSS/EBV(-) vs MSI-H), interaction (sex and MSI status); [3] Post-therapeutic factors included: Sex, age (≥55 vs <55 yrs), localization (proximal vs non-proximal), Laurén subtypes (intestinal vs non-intestinal), neoadjuvant chemotherapy (yes vs no), (y)pT (pT1-pT4), (y)pN, M-category, R-category, MSI status (MSS/EBV(-) vs MSI-H), interaction (sex and MSI status); [4] TNM classification according to 7th Edition UICC.

Table S6. Multivariable analysis of survival including the interaction of sex and MSI status and pre- and posttherapeutically available clinical factors in MSI-H and MSS/EBV(–) patients treated with neoadjuvant CTx (n = 384).

	HR	95% CI	*p*-Value [1]
**CTx patients: Pretherapeutic factors [2]**			
**Interaction (Sex and MSI status)**	0.14	0.02-1.01	0.051
**CTx patients: Posttherapeutic factors [3]**			
**R-category**			
R0	1 ref.	-	0.001
R1	1.71	1.23-2.37	
**ypN[4]**			
ypN0	1 ref.	-	<0.001
ypN1	2.42	1.68-3.50	
**Metastasis status**			
Negative	1 ref.	-	<0.001
Positive	1.73	1.27-2.35	
**ypT[4]**			0.019
ypT0	0.17	0.02-1.25	0.082
ypT1	0.32	0.11-0.90	0.031
ypT2	0.54	0.30-0.99	0.047
ypT3	0.64	0.46-0.89	0.008
ypT4	1 ref.	-	-
**Interaction (Sex and MSI status)**	0.16	0.02-1.14	0.067

ref., reference; MSS, microsatellite stable; EBV(–), Epstein-Barr virus negative; MSI-H, high microsatellite instability, HR; Hazard ratio; CI, confidence interval. [1] Wald-Test of Hazard Ratio; [2] Pre-therapeutic factors include: Sex, age (≥55 vs <55 yrs), localization (proximal vs non-proximal), Laurén subtypes (intestinal vs non-intestinal), clinical tumor stage (cT2 vs cT3/4), MSI status (MSS/EBV(-) vs MSI-H), interaction (sex and MSI status); [3] Post-therapeutic factors included: Sex, age (≥55 vs <55 yrs), localization (proximal vs non-proximal), Laurén subtypes (intestinal vs non-intestinal), (y)pT (pT1-pT4), (y)pN, M-category, R-category, MSI status (MSS/EBV(–) vs MSI-H), interaction (sex and MSI status); [4]TNM classification according to 7th Edition UICC.

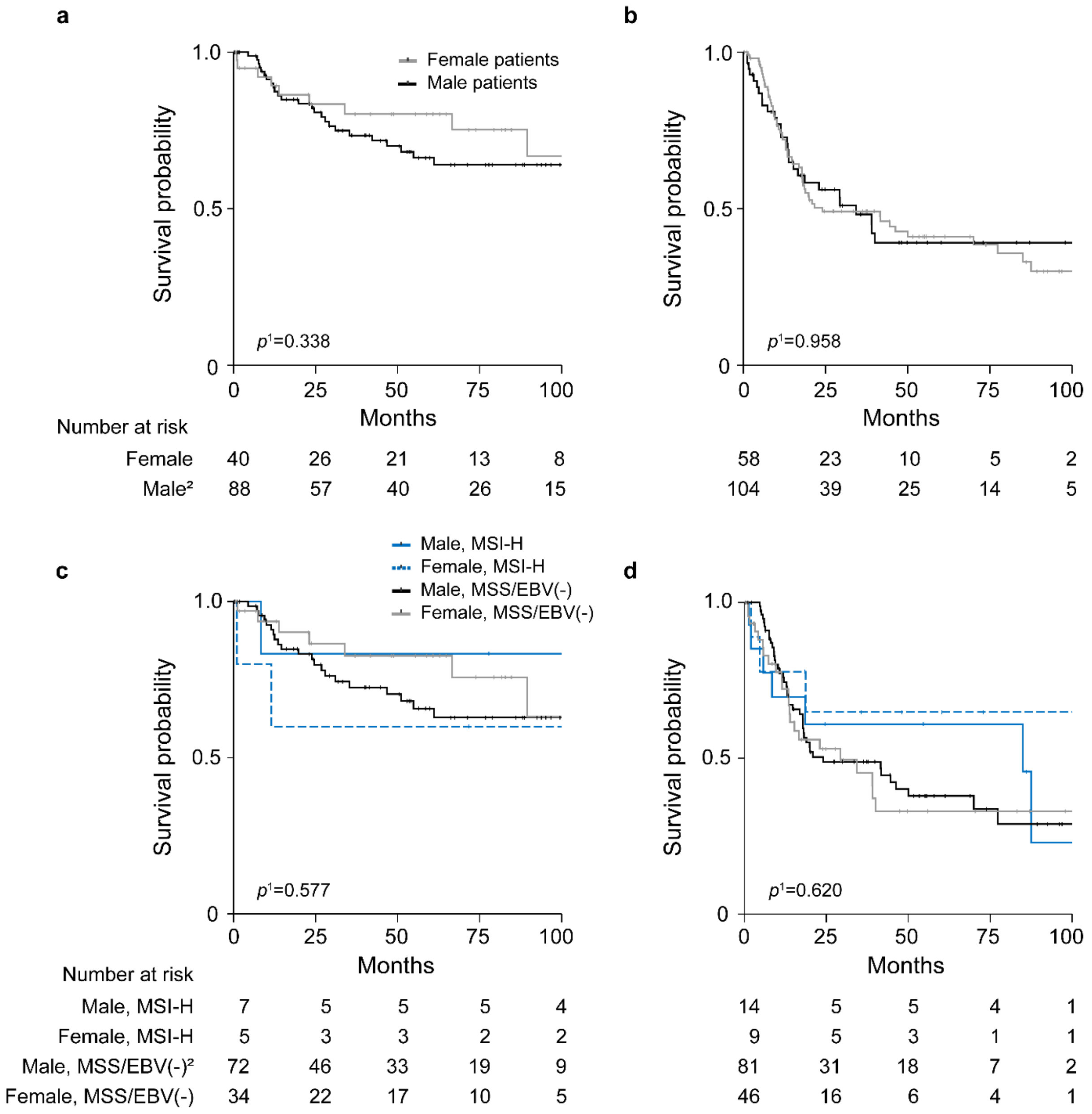

Figure S1. Patients' survival in the non-CTx group stratified according to clinical tumor stage. Kaplan-Meier curves of patients treated with surgery alone stratified according to the clinical tumor stage (cT) and discrimination by sex in cT2 (**a**) and cT3/cT4 (**b**) patients and by sex and MSI status in cT2 (**c**) and cT3/cT4 (**d**) patients are shown. [1] Cox-regression; [2] For one male patient with a MSS/EBV(-) tumor no cT data were available.

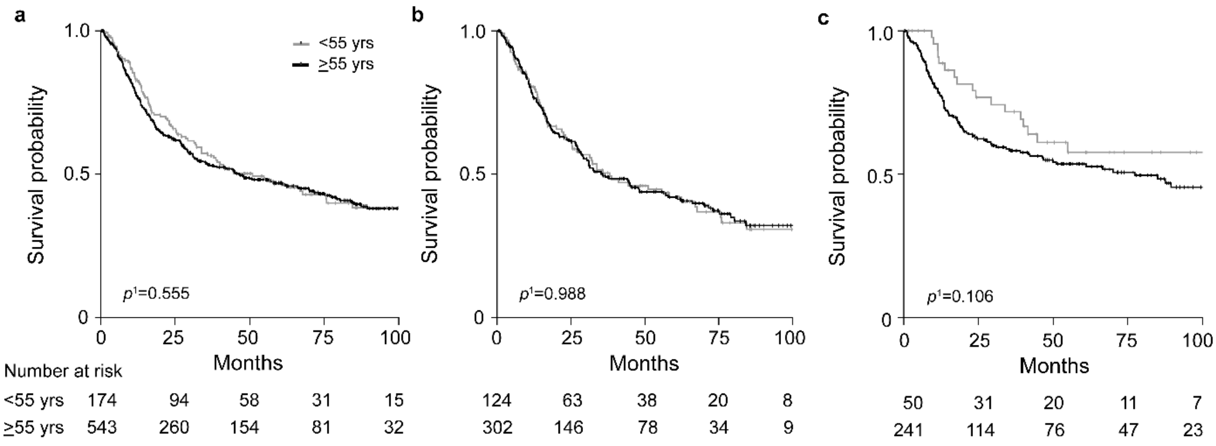

Figure S2. Discrimination of patients' survival by age. Kaplan-Meier curves of younger (<55 yrs) and older ($\geq$55 yrs) patients are shown. All patients (**a**), patients treated with neoadjuvant CTx (**b**) and patients treated with surgery alone (**c**). [1] Cox-regression.

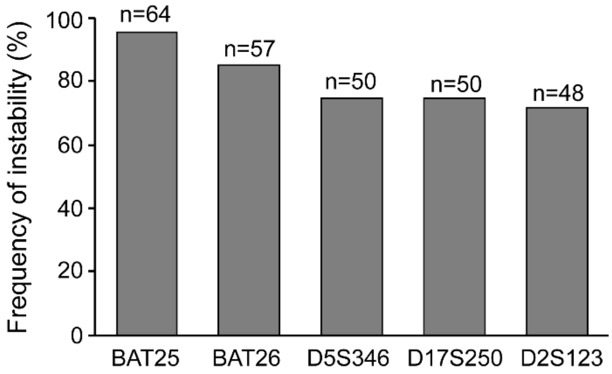

Figure S3. Frequency of instabilities at five microsatellite markers included in the Bethesda panel in MSI-H tumors (n = 67).

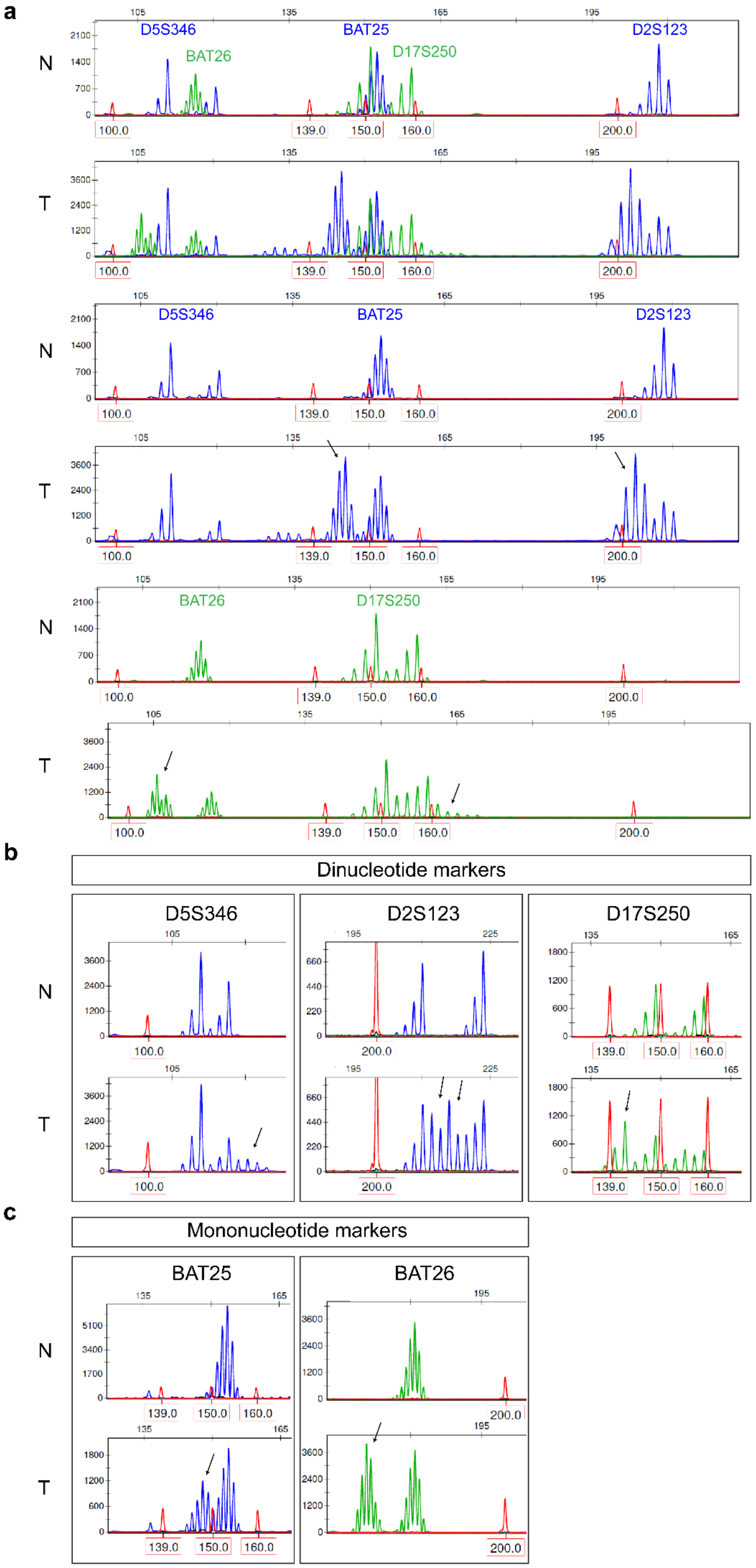

Figure S4. Capillary electrophoresis results of MSI analysis using the five microsatellite markers included in the Bethesda panel. Microsatellite patterns at three dinucleotide (D5S346, D17S250, D2S123) and two mononucleotide (BAT25, BAT26) repeat markers are shown exemplarily for the multiplex PCR of a tumor specimen (T) and its corresponding non-tumorous

tissue (N). Multiple additional alleles and distinct shifts occur at analyzed microsatellite loci in the tumor sample in comparison to the normal tissue as indicated with arrows. The upper two panels show an overview about an electropherogramm of all five markers. To clearly show the instabilites, the FAM (blue) and HEX (green) fluorescence-tagged fragments are shown separately in the respective panels below (**a**). Examples of MSI patterns from different tumor specimens and their corresponding non-tumorous tissues are shown for the dinucleotide markers (**b**) and the mononucleotide markers (**c**) respectively. The red peaks are internal size standards. The x-axis displays the size in bases and the y-axis the respective fluoresence intensity.
